# Photoacoustic Laser System for Food Fraud Detection

**DOI:** 10.3390/s21124178

**Published:** 2021-06-18

**Authors:** Luca Fiorani, Florinda Artuso, Isabella Giardina, Antonia Lai, Simone Mannori, Adriana Puiu

**Affiliations:** Diagnostics and Metrology Laboratory, Nuclear Fusion and Safety Technologies Department, ENEA, Via Enrico Fermi 45, 00044 Frascati, Italy; florinda.artuso@enea.it (F.A.); isabella.giardina@enea.it (I.G.); antonia.lai@enea.it (A.L.); simone.mannori@enea.it (S.M.); adriana.puiu@enea.it (A.P.)

**Keywords:** quantum cascade laser application, laser spectroscopy, photoacoustic technique, agrofood chain, food fraud

## Abstract

Economically motivated adulterations of food, in general, and spices, in particular, are an emerging threat to world health. Reliable techniques for the rapid screening of counterfeited ingredients in the supply chain need further development. Building on the experience gained with CO_2_ lasers, the Diagnostic and Metrology Laboratory of ENEA realized a compact and user-friendly photoacoustic laser system for food fraud detection, based on a quantum cascade laser. The sensor has been challenged with saffron adulteration. Multivariate data analysis tools indicated that the photoacoustic laser system was able to detect adulterants at mass ratios of 2% in less than two minutes.

## 1. Introduction

Food fraud is a complex problem of the world market: the globalization of trade and the lack of shared standards for quality control pose serious threats to final consumers. Interpol and Europol conduct a joint operation to combat economically motivated adulteration [1], estimating that the world food market is about USD 300 billion, of which 15% is online, a sales platform on which it is easier to fool customers.

Many techniques have been deployed in this war against crime but, unfortunately, it is still true what Jeffrey C. Moore wrote in 2011: “the paucity of analytical detection methods means that the safety of counterfeit foods is in the hands of fraudsters” [2]. The same author recommended the development of “non-targeted approaches for making identifications, using rapid spectral techniques combined with chemometrics or multivariate data-analysis tools”, pointing out that “Identification tests that work in compendial and rapid industrial settings remain to be developed” and emphasizing the importance of “rapid confirmatory methods; verification of abnormality is crucial, before removal of an ingredient from the supply chain”.

In this framework, the Diagnostics and Metrology Laboratory (FSN-TECFIS-DIM) of the Italian National Agency for New Technologies, Energy and Sustainable Economic Development (ENEA) [3] applies laser photoacoustic spectroscopy (LPAS) [4] to the quality control of the agro-food chain [5].

In a typical LPAS system, a laser beam is modulated at an audio frequency and injected into a resonant cell where it hits the investigated sample that absorbs the incident radiation. As a consequence, the part of the sample irradiated by the beam experiences a rise in temperature and volume, thus producing a pressure wave. In general, the sound detection subsystem is made of a microphone connected with a lock-in amplifier synchronized with the modulator. The output signal is proportional to the sample absorption and typical experiments are conducted in the “fingerprint region”, a large band of the infrared (IR) spectrum where many organic compounds can be identified. In some sense, LPAS is similar to IR spectroscopy, but with the important advantage of an unrivaled power of the radiation source (laser vs. lamp).

The pioneering studies carried out at ENEA with CO_2_ lasers showed that LPAS has the following advantages:

- Rapidity: a food fraud can be detected in a few minutes.
- Sensitivity: CO_2_ laser-based LPAS systems—due to the high power of the radiation source—can be sensitive, especially in the case of gas absorption.
- Specificity: the absorption lines are a “fingerprint” of the compound to be detected.
- Simplicity: neither sample pretreatment nor chemical reagents are required.
- Repeatability: measurements can be repeated simply because samples are not affected by radiation.
- In situ measurement: LPAS systems are deployable in food production, storage and distribution sites, departments of public health and custom borders.
- Uncomplicated sampling: the operation requires only inserting a small fragment of food inside the cell.
- Ease of use: LPAS systems can be designed for use by non-expert personnel.
- Cost-effectiveness: if compared to other analytical techniques (in terms of both initial investment and operating costs—the latter are practically zero because no reagents or spare parts are needed).

Unfortunately, it is hard to miniaturize a CO_2_ laser and, as a consequence, these systems are not easily portable. Another disadvantage of these radiation sources is that they have only some emission lines in a limited spectral range [6,7].

To overcome these drawbacks, it has been decided to replace CO_2_ lasers with quantum cascade lasers (QCLs) [8]: although their power is still lower, implying a possible worsening of the sensitivity, their wavelength can be continuously tuned in a large spectral range. This latter characteristic is very important for non-targeted approaches and can compensate for the power reduction. Moreover, they are robust and small, allowing one to develop a portable system for rapid detection of food fraud in industrial settings.

Once developed, the system has been challenged with the frequent economically motivated adulteration of saffron, having in mind that “herbs and spices” are an important sector of the food market, valued at about USD 4 billion [9], and saffron is the fourth most common target for adulteration reported in the literature [10].

Saffron is part of *Crocus sativus*, commonly known as saffron crocus, a flower with three stigmas that are collected and dried. Ground saffron is often cut with tartrazine—a synthetic yellow dye used as a food coloring—and turmeric powder—a yellow spice obtained from the rhizome of *Curcuma longa*, a flowering plant known as turmeric [11].

Crocins (16–28% mass ratio), picrocrocin (7–16%) and safranal (0.1–0.6%) give saffron its color, taste and aroma, respectively. Picrocrocin has been found only in saffron and is regarded as its molecular marker [12]. At present, according to ISO 3632 [13], the standard control of saffron authenticity is ultraviolet–visible (UV–vis) spectrophotometry of water-extracted samples [14]. Typically, aqueous or organic extracts of ground saffron are prepared and submitted to magnetic stirring or ultrasound-assisted extraction, then they are filtered with a porosity of 0.45 µm and eventually their spectral characteristics are measured by scanning from 200 to 700 nm using a spectrophotometer: absorbance at 257, 330 and 440 nm is related to picrocrocin, safranal and crocins, respectively. Unfortunately, it has been shown [15] that picrocrocin and safranal are overestimated by this technique because other compounds absorb at 257 and 330 nm. High-performance liquid chromatography with a diode-array detector (HPLC-DAD) of water-extracted samples [16] and gas chromatography-mass spectrometry (GC-MS) with different extraction and injection systems [17] have been suggested as an alternative. In HPLC-DAD: “Twenty microliters of … saffron extracts … were injected into an … HPLC chromatograph … equipped with a 150 mm × 4.6 mm i.d., 5 µm … column that was equilibrated at 30 °C. The eluents were water (A) and acetonitrile (B) with the following gradient: 20% B, 0–5 min; 20–80% B, 5–15 min; and 80% B, 15–20 min. The flow rate was 0.8 mL/min. The DAD detector … was set at 250, 330, and 440 nm for picrocrocin, safranal and crocetin ester detection, respectively”. In GC-MS: “The mass detector was an ion trap spectrometer equipped with an electrospray ionization interface … The full scan mass covered the range from *m*/*z* 200 up to 1500. Collision-induced fragmentation experiments were performed in the ion trap using helium as a collision gas … All mass spectrometry data were recorded in the negative ion mode”. In addition to UV–vis, IR and nuclear magnetic resonance (NMR) spectroscopies are also used for saffron quality assessment [18]. Unfortunately, spectroscopic methods, HPLC-DAD and GC-MS are complex, expensive and slow, require preparation and destruction of the sample, have to be operated by qualified personnel (research scientists, laboratory technicians, trained policemen…) and cannot be deployed in food production, storage and distribution sites, departments of public health and custom borders.

## 2. Materials and Methods

### 2.1. Photoacoustic Laser System

**Figure 1:**
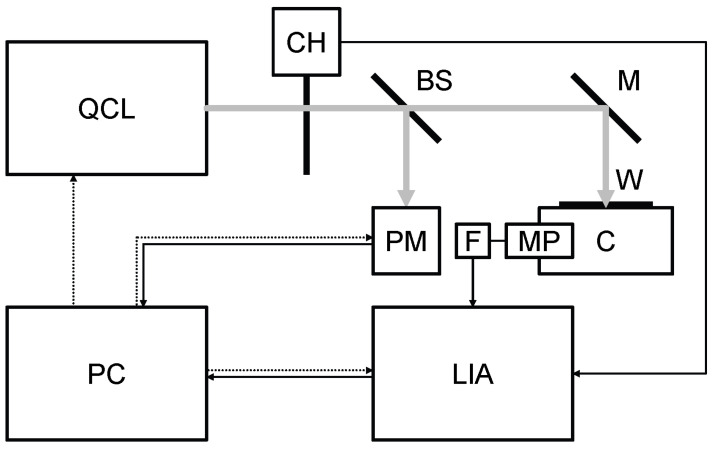
Block diagram of the photoacoustic laser system. BS: beam splitter, C: photoacoustic cell, CH: chopper, F: active low pass filter, LIA: lock-in amplifier, M: mirror, MP: microphone, PC: personal computer, PM: power meter, QCL: quantum cascade laser, W: window. Grey continuous line: laser beam, black continuous line: signal, black dotted line: control.

A block diagram of the photoacoustic laser system is given in Figure 1. Its main elements are listed in Table 1. The continuous wave emitted by the QCL (Table 2) is modulated at the resonance audio frequency of the cell (215 Hz) by the chopper and sent by a mirror and through a window to the food sample (of the order of 50 mg) inside the photoacoustic cell. The sound is detected by the microphone coupled with the lock-in amplifier through an active low pass filter (gain: 10, cutoff: 3 kHz). The beam splitter sends 18% of the laser beam to the power meter and 70% to the mirror (12% is absorbed by the beam splitter). Peak power is more than 50 mW, mirror reflectance is nearly 99% and window transmittance is practically 100%. The lock-in amplifier is synchronized with the audio frequency by a trigger signal coming from the chopper. A personal computer is interfaced with QCL, power meter and lock-in amplifier. At first, the QCL is tuned at the desired wavelength. Then, the lock-in amplifier measures the voltage coming from the microphone and passing through the active low pass filter. At the same time, the power meter measures the power emitted by the QCL. These operations are repeated for each wavelength of the spectrum.

The experiment is controlled by a MATLAB application developed in our laboratory. The lock-in amplifier communicates through an RS-232 serial interface with a USB converter and open ASCII protocol compatible with MATLAB. QCL and power meter are connected to a USB hub which—in turn—is linked to the personal computer. The output file uses a standard ASCII format, which is easily readable and can be imported into MATLAB, Excel or other post-processing applications. Its name is automatically generated, to make it impossible to accidentally overwrite previous measurements. The graphic user interface (GUI) requires the three parameters of the spectrum (wavelength minimum, maximum and step) and the number of measurements to be repeated at each wavelength (measurement duration: 1 s). Any point of the graphic of photoacoustic signal vs. wavelength (LPAS spectrum) is calculated by dividing the average of lock-in amplifier voltages by the average of laser powers (the average is calculated over the repeated measurements).

First, the photoacoustic laser system has been thoroughly tested with a standard material (activated carbon): more information on laser power vs. wavelength curve, cell frequency response and system linearity can be found here [8]. Then, as previously said, it has been challenged with the realistic simulation of two important food frauds [19]: saffron adulterated with both tartrazine and turmeric.

The samples have been prepared starting from the following Sigma-Aldrich standards:

- SA (saffron): Saffron S8381;
- TA (tartrazine): Tartrazine 03322;
- TU (turmeric): Curcumin, Curcuma longa 239802.

In the following we will name:

- “component”, every single standard;
- “contaminant”, both tartrazine and turmeric;
- “mixture” a sample obtained mixing a contaminant with saffron.

Tartrazine and turmeric were already powder, while saffron stigmas delivered by Sigma-Aldrich have been ground to prepare the mixtures with a ball pestle impact grinder. In real cases, grinding is not necessary: saffron authenticity is assessed simply by inserting about 50 mg of the commercial product under investigation inside the photoacoustic cell.

Mixtures with TA/SA and TU/SA mass ratios of 2%, 5%, 10% and 20% have been made, using a high accuracy analytical balance for the weighing procedure, to calibrate [20] the photoacoustic laser system for medium-low contaminant mass ratios. This adulteration range has been chosen both to mimic frequent frauds [19] and to challenge the system at limits of detection similar to electronic nose (10%) [21], Fourier-transform IR spectroscopy (FTIR) (5%) [22], gas chromatography (GC) (5%) [23], HPLC (2%) [24], laser-induced breakdown spectroscopy (LIBS) (2%) [25] and NMR (20%) [26].

### 2.2. Data Analysis

All the spectra shown in this paper have been obtained as follows:

1. The QCL scanned the wavelengths from 8.75 to 11.00 μm with a step of 0.025 μm (91 wavelengths, corresponding to a minute and a half).
2. The lock-in amplifier and the power meter measured photoacoustic signal (V) and laser power (W), respectively. Each measurement lasted 1 s and was repeated 10 times and the 10 measurements of signal and power were averaged.
3. The LPAS signal (V/W) is given by the ratio of those average measurements (thus normalizing the photoacoustic signal by the laser power).
4. Each QCL scan was repeated 10 times and the 10 scans were averaged.
5. The final spectrum is the result of a third-order 9-point Savitzky–Golay filter applied to that average scan.

**Figure 2:**
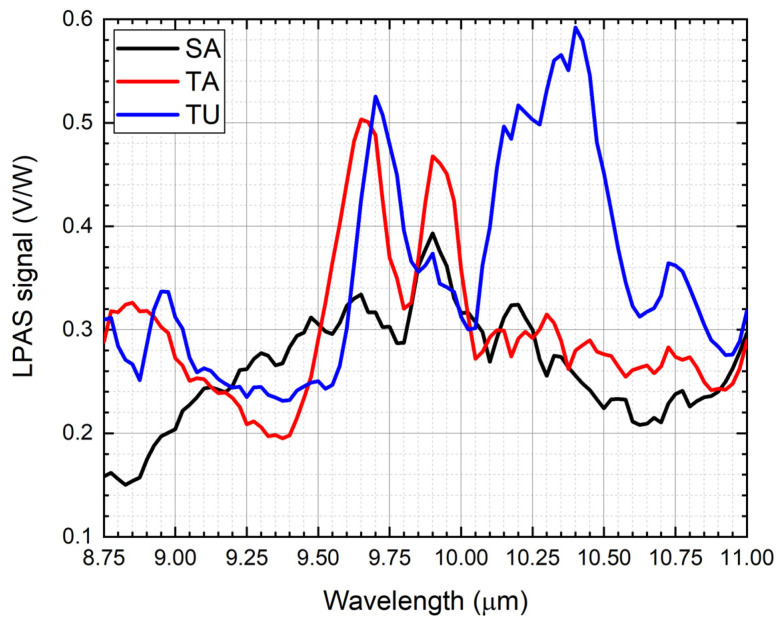
Photoacoustic spectra of saffron, tartrazine and turmeric.

Figure 2 shows the spectra of saffron, tartrazine and turmeric. The more important peaks are at:

- 9.80–10.10 μm for saffron.
- 9.50–9.80 μm and 9.80–10.10 μm for tartrazine.
- 9.60–9.85 μm and 10.05–10.60 μm for turmeric.

**Figure 3:**
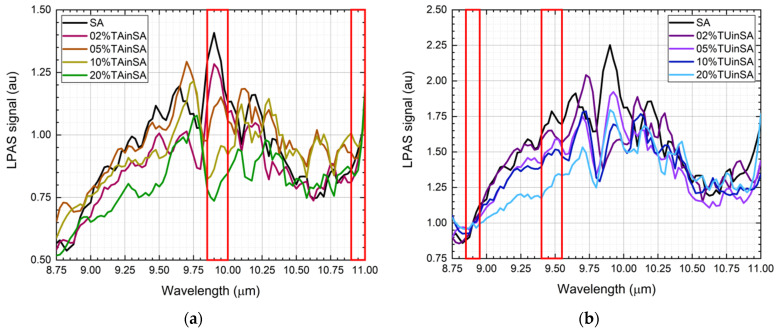
(**a**) Photoacoustic spectra of saffron, 2% of tartrazine in saffron, 5% of tartrazine in saffron, 10% of tartrazine in saffron and 20% of tartrazine in saffron. The spectra have been normalized in the 10.90–11.00 μm range (right red box). Absorption in the 9.85–10.00 μm range (left red box) drops while the tartrazine mass ratio increases. (**b**) Photoacoustic spectra of saffron, 2% of turmeric in saffron, 5% of turmeric in saffron, 10% of turmeric in saffron and 20% of turmeric in saffron. The spectra have been normalized in the 8.85–8.95 μm range (left red box). Absorption in the 9.40–9.55 μm range (right red box) drops while the turmeric mass ratio increases.

Only average spectra have been shown in Figure 2. The precision of each point of all the spectra has been calculated from the standard deviation. It ranges from 0.5% to 5% and is 2.0% on average. Looking at them, one could expect an easy disentanglement of the contribution of each component in the spectra of mixtures, at least when the contaminant mass ratio is high. Unfortunately, Figure 3 shows that the spectra of the mixtures are largely different from the linear combination of the spectra of the components, e.g., let us compare saffron and 20% of tartrazine in saffron. The mixture indeed has an important peak at 9.50–9.80 μm, where only tartrazine effectively absorbs, but at 9.80–10.10 μm, where both saffron and tartrazine effectively absorb, a local minimum appears, suggesting that chemical and physical nonlinear processes take place in the mixture. Similar considerations can be made for all mixtures.

Bearing in mind that the final purpose of the photoacoustic laser system is the quantification of the mass ratio of a contaminant from its spectrum, regardless of the chemical and physical processes that drive its shape, three data analysis methods have been applied to carry out that quantification:

1. Absorption ratio at two wavelengths.
2. Principal component analysis (PCA).
3. Partial least squares regression (PLS).

The rationale for applying such a simple method as absorption ratio at two wavelengths relies on the desire to find a spectral feature that varies almost linearly with the contaminant mass ratio, to be confident that more sophisticated linear methods will be successful. According to [20]: “With multivariate calibration, more than one wavelength is used allowing correction of spectral interferences and other matrix effects such as chemical interactions … Depending on the degree of nonlinearities, linear multivariate regression may be able to correct the nonlinear deviations”.

The application of linear multivariate regression has been preceded by PCA, to check if it was possible to ascertain principal components from the experimental data. PCA has been applied to the experimental data with OriginPro 2020 [27].

According to [20], PLS is the most promising linear multivariate regression if nonlinear processes are present, as in our case. Additionally, PLS has been applied to the experimental data with OriginPro 2020 [27].

## 3. Results and Discussion

### 3.1. Absorption Ratio at Two Wavelengths

**Figure 4:**
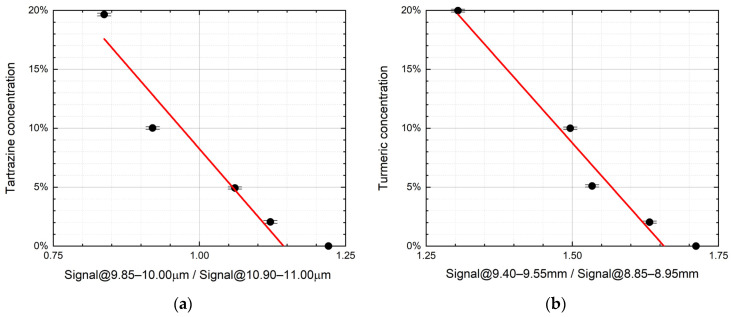
(**a**) Tartrazine mass ratio vs. the normalized LPAS signal in the 9.85–10.00 μm range, i.e., the ratio of the average LPAS signals in the 9.85–10.00 μm and 10.90–11.00 μm ranges (r^2^ = 0.93). (**b**) Turmeric mass ratio vs. the normalized average LPAS signals in the 9.40–9.55 μm range, i.e., the ratio of the average LPAS signals in the 9.40–9.55 μm and 8.85–8.95 μm ranges (r^2^ = 0.97). The error bars depend on the weighing accuracy.

After a careful inspection of the spectra, it has been observed that:

- For the saffron–tartrazine mixtures, once spectra have been normalized in the 10.90–11.00 μm range (“normalization range” of tartrazine), the absorption in the 9.85–10.00 μm range (“sensible range” of tartrazine) decreases while the tartrazine mass ratio increases (Figure 4a).
- For the saffron–turmeric mixtures, once spectra have been normalized in the 8.85–8.95 μm range (“normalization range” of turmeric), the absorption in the 9.40–9.55 μm range (“sensible range” of turmeric) decreases, while turmeric mass ratio increases (Figure 4b).

An attempt has been made to linearly relate the contaminant mass ratio with the normalized average LPAS signal in the respective sensible range (Figure 4).

Although the residual of the predicted contaminant is quite large (up to more than 4% for no tartrazine in saffron), the linear correlation is surprisingly good if compared with the nonlinear behavior of spectra. It is not unexpected that the prediction of both linear models is particularly unsatisfactory for pure saffron because, only in this case, the spectrum is not affected by the chemical and physical processes that distort all the mixture spectra.

### 3.2. PCA

**Figure 5:**
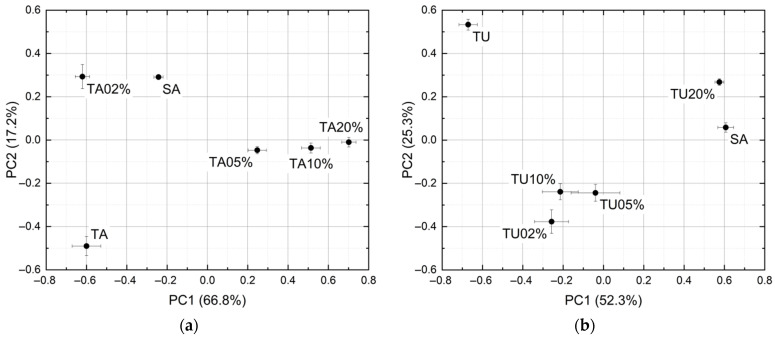
PCA score plots of the spectra of: (**a**) Tartrazine and saffron. (**b**) Turmeric and saffron.

PCA has been applied to non-averaged spectra. Due to nonlinear effects, the points of the score plot corresponding to the mixtures are not in the segment linking the points corresponding to pure saffron and pure contaminant (Figure 5). Nevertheless, the principal components disentangle the different samples. To have better readability, instead of plotting the ten points of the cluster, the average and standard deviation of their PC1 and PC2 coordinates have been drawn. The explained variances are 84.0% and 77.6% for tartrazine and curcumin mixtures, respectively.

### 3.3. PLS

**Figure 6:**
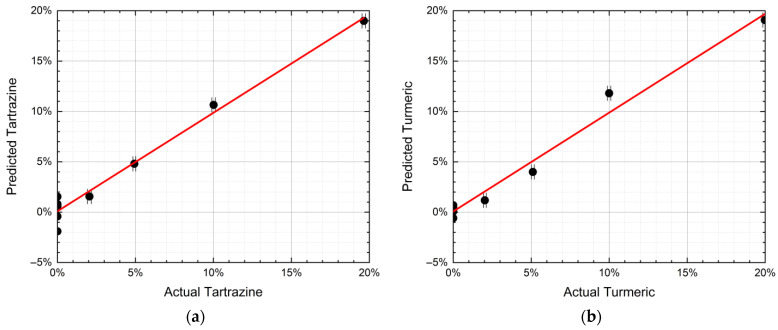
(**a**) PLS-predicted tartrazine mass ratio vs. the actual mass ratio (r^2^ = 0.98). (**b**) PLS-predicted turmeric mass ratio vs. the actual mass ratio (r^2^ = 0.98). The error bars depend on the weighing accuracy.

The calculation converged with six factors, thus explaining 99.5% of the variance for x (effects) variables and 98.4% of the variance for y (responses) variables. The discrepancy between predicted and actual contaminant mass ratio is shown in Figure 6 both for tartrazine and turmeric. The 2% contamination is detected, both for tartrazine and turmeric. The discrepancy between predicted and actual mass ratios is below 2% for all mass ratios in both cases (on average 0.80% for tartrazine and 0.76% for turmeric).

### 3.4. Discussion

Nonlinear effects are noticeable. As has already been mentioned, the spectra of the mixtures are largely different from the linear combination of the spectra of the components and, in the PCA, the points of the score plot corresponding to the mixtures are not in the segment linking the points corresponding to pure saffron and pure contaminant. Nevertheless, all adulterated samples are discriminated from pure saffron in the score plots, leading to a correct classification rate of 100% down to saffron + 2% tartrazine and saffron + 2% turmeric. In this case study, the photoacoustic laser system reached a selectivity of 100%.

Nonlinearities can be induced by matrix effects linked to physical (optical) and chemical interactions between saffron and contaminant. One possible cause can be searched in the different grain sizes of the samples that—as it has already been observed [22,28]—can affect the spectra features. Nevertheless, PLS seems able to correct the nonlinear deviations and to predict the contaminant mass ratio with a sensitivity of 2% and an accuracy of 1%. These measurements are traceable in the sense that they have been carried out on reference standards (certified by Sigma-Aldrich).

Although these results are preliminary, they are encouraging if compared with the performances of other systems:

- Electronic nose, sensitivity: 10%, correct classification rate: 62.5% (saffron + 25% corn stigmas), 100% (saffron + 10% safflower) [21];
- FTIR, sensitivity: 5%, correct classification rate: 99% (saffron + 5% adulterant, where adulterant is buddleja, calendula, gardenia, safflower, saffron stamens and turmeric) [22];
- GC, sensitivity: 5%, correct classification rate: 100% (saffron + 5% marigold and saffron + 5% turmeric) [23];
- HPLC, sensitivity: 2%, correct classification rate: 100% (saffron + 50% adulterant, where adulterant is marigold, safflower and turmeric) [24];
- LIBS, sensitivity: 2%, correct classification rate: 100% (saffron + 5% adulterant, where adulterant is marigold, safflower and turmeric) [25];
- NMR, sensitivity: 20%, correct classification rate: 100% (saffron + 20% adulterant, where adulterant is gardenia, safflower, saffron stamens and turmeric) [26].

This study provided an overall confirmation of the advantages of LPAS already stated in the introduction for CO_2_ laser-based systems; in particular, rapidity, simplicity, repeatability, in situ measurements, uncomplicated sampling, ease of use and cost-effectiveness: none of the above-mentioned competitor methods simultaneously has all of them. Nevertheless, although these preliminary results indicate that the photoacoustic laser system is comparable to other technologies—at least in this case study—laboratory instruments will likely remain unrivaled in terms of sensitivity and specificity, especially when lower limits of detection are required: in the hard battle against food fraud, the photoacoustic laser system could be the front line, providing a first quick indication that a supply is suspicious and further analysis by a laboratory instrument is needed. The simultaneous deployment of both technologies will merge their advantages, thus improving efficiency, rapidity and affordability in combating this crime. The QCL-based photoacoustic laser system promises also to be able to detect many food frauds for at least three reasons: (1) it investigates a larger wavelength range compared to its forerunner, the CO_2_ laser-based system, which sensed several adulterations (see [3] and the references contained therein); (2) a preliminary setup of the QCL-based photoacoustic laser system was able to discriminate different kinds of rice [8]; (3) ongoing data analysis on other agro-food adulterations suggests that the present instrument can detect them. In a nutshell, thanks to its advantages, the photoacoustic laser system is a good candidate for rapid and non-targeted detection of food fraud in industrial settings, large retailers and other everyday life application scenarios. A good strategy to achieve this goal could be to improve the human–machine interface and to install the system in a cart so that it can be operated by unqualified personnel and deployed in food production, storage and distribution sites, departments of public health and customs borders. Moreover, more data have to be acquired (e.g., increasing the number of mass ratios) and the photoacoustic laser system has to be applied to the rapid and non-targeted detection of food fraud in other relevant case studies.

## 4. Conclusions

Building on previous experiences on the application of LPAS to the quality control of the agro-food chain, a new QCL-based compact photoacoustic laser system has been developed.

The focus on this stage of the research was on the development of a portable sensor for rapid and non-targeted detection of food fraud in everyday life application scenarios, thanks to the advantages of LPAS compared to other technologies (rapidity, simplicity, repeatability, in situ measurements, uncomplicated sampling, ease of use and cost effectiveness). To prove the concept, saffron contamination by tartrazine or turmeric has been chosen as a case study. Although the spectra showed nonlinear effects, in two spectral zones the signal was inversely proportional to the amount of contaminant (one zone relative to tartrazine and the other one to turmeric). Each sample has been measured ten times and PCA was able to discriminate pure spice and contaminants, as well as contaminant-spice mixtures down to a mass ratio of 2%. Additionally, the prediction of the contaminants mass ratio by PLS was possible. Although it would have been desirable to increase the numbers of both measurements and mass ratios of the samples, all these results taken together indicate that the photoacoustic laser system has the capability to detect an important food fraud of “herbs and spices” in a minute and a half with a sensitivity of 2% and an accuracy of 1%, provided its spectra are analyzed with multivariate analysis.

The next stage of the research will be to challenge the sensor with other case studies, acquiring a large number of spectra for many mass ratios of different adulterants. Moreover, the spectra should be enlarged, to easily find a spectral band where the peaks of adulterant and unadulterated food do not overlap. In the case of the QCL used in this study, more modules can be implemented, thus extending the wavelength range from 6.0 to 11.11 µm. In the meantime, the human–machine interface has to be improved and the optoelectromechanical systems have to be installed in a cart, so that the prototype can be operated by unqualified personnel and deployed in food production, storage and distribution sites, departments of public health and customs borders.

## Figures and Tables

**Table 1:** Main elements of the photoacoustic laser system.

Element	Manufacturer	Model
BS	Thorlabs	WG71050
C	ENEA ^1^	N.A.
CH	Stanford Research Systems	SR540
F	Hewlett-Packard	5489A
LIA	Stanford Research Systems	SR530
M	Thorlabs	PF10-03-M02
MP	Knowles	EK23024000
PC	Asus	X55C
PM	Gentec-EO	XLP12-1S-H2-DO
QCL	DRS Daylight Solutions	MIRcat-1200
W	Thorlabs	WG71050-E4

^1^ The cell has been manufactured at ENEA.

**Table 2:** Main specifications of the QCL.

Parameter	Value
Wavelength range	8.33–11.11 µm
Linewidth	100 MHz
Wavelength accuracy	1 cm^−1^
Average power	60 mW
Power stability	3%
Spatial mode	TEM_00_
Beam divergence	4 mrad
Beam pointing stability	2 mrad
Spot size	2.5 mm
Polarization	Vertical 100:1

## Data Availability

The data presented in this study are available on request from the corresponding author. The data are not publicly available due to ENEA Research Data Policy.
